# Of teachers and students…

**DOI:** 10.4103/0971-5851.73587

**Published:** 2010

**Authors:** Sudeep Gupta

**Affiliations:** *Editor-in-Chief, Tata Memorial Hospital, Mumbai, India. E-mail: sudeepgupta04@yahoo.com*

It was the best of times, it was the worst of times, it was the age of wisdom, it was the age of foolishness… we had everything before us, we had nothing before us…

*Charles Dickens* in “A Tale of Two Cities”

Qualified medical professionals in academic institutions are in the unique position of donning several garbs simultaneously. They are doctors whose primary role is to bring health to patients. They are teachers who have the responsibility of passing knowledge, skills, ethics and some of their wisdom to the next generation. Many are also researchers who spend a lifetime asking and answering questions and mentoring young aspirants in the art and science of research. And lastly, qualified medical professionals remain literal students for long years in their careers.

Are individuals able to juggle these roles effectively? Has the quality of communication changed over time and impacted these avatars? The preceding decades have seen changes in human interaction like no other epoch in history. Advances in electronics, computing, miniaturization, etc. have placed enormous resources of information and communication within individual reach. The medical universe has not remained unaffected. Objective and updated information is now available to medical students outside the confines of scholarly tomes. The current generation of students accesses editorials, reviews and other journal articles more than its peers in the past. The teachers also use these and other resources in an ever more beguiling array of presentations. There is greater emphasis on specialization – young doctors now see themselves as cardiologists even before they have the self image of a physician. There is far greater reliance on the results of investigations using sophisticated technology, which of course is good for diagnostic and therapeutic precision. The result is (probably) better informed individuals when they exit medical schools. Faculty members increasingly emphasize their roles as researchers and star speakers at glitzy conferences; teaching, especially bedside encounters with patients, is often incidental.

Has something been lost? The long hours spent in rounding on patients, with experienced physicians, lent itself not only to fatigue but also to keen observation, learning the art of empathetic communication and elicitation of physical signs. Bonds were forged that sometimes lasted a lifetime. There was emphasis on integration of data to arrive at clinical impressions and differential diagnoses. There was also, of course, the tyrannical professor whose word was law, fortunately becoming rarer. Considerable stress was placed on acquisition of well-rounded clinical skills in many disciplines during compulsory internship, now often replaced by seeking mastery over multiple choice questions. There were no short messages, mobiles, tweets, mails, profiles and pop-ups to distract. I sometimes wonder about the average attention span of successive generations on any activity. Concentration and single-minded focus, essential inputs in some of the greatest human accomplishments, have become casualties of much vaunted multitasking skills. I may be biased, but I have the impression that thoughts and ideas are increasingly fragmented and tunneled in the modern physician, as indeed in the modern human. The fragmentation manifests itself in the way medical teachers and students now interact.

*The Guru-Shishya* tradition in medicine was a valuable part of a system that resulted in a generation of medical leaders who were also dedicated teachers. It needs to be reinvented and preserved. The advances in technology and specialization are complementary, not antithetical. There is something about the first words that a child learns from her caregiver – no technology will ever replace the two-way human transfer of…not only language…but a range of associated emotions. But then again, I perhaps lament too much the passing of a bygone era – my own existential angst.

